# A closed form shape function describing 3D settlement field around a deep excavation in sand

**DOI:** 10.1038/s41598-022-22003-8

**Published:** 2022-11-02

**Authors:** Gianpiero Russo, Marco Valerio Nicotera

**Affiliations:** Department of Civil, Architectural and Environmental Engineering, Università di Napoli Federico II, Via Claudio, 21, 80125 Naples, Italy

**Keywords:** Civil engineering, Computer science

## Abstract

Soil movements produced around deep excavations are one of the main issues to be addressed during design and construction of underground structures in urban environment. Several methods to predict ground movements are currently available. Semi-empirical methods correlate displacements and simple geometrical features of the excavation; these methods predict separately transversal and longitudinal settlement troughs or at least provide a conservative envelope of them and they are mainly based on empirical data of excavations in clay. Numerical methods based on the solutions of FEM or DFM models usually provide prediction of the green field subsidence. In this paper existing 2D semi-empirical methods are first shortly reviewed. Some of these methods are then modified and combined into a new 3D analytical description of the subsidence trough around a deep excavation. The proposed two variable subsidence function depends on several parameters: four independent parameters defining the overall shape and the maximum settlement acting as a scale parameter. Settlement fields reproduced by a 3D FEM nonlinear parametric study of deep excavations inspired by real case studies are thus presented and discussed. The newly proposed 3D analytical description is applied to fit the FEM results and some fundamental relationships among geometrical features of the excavation pit and the 3D shape function parameters are identified. These relationships are validated via the application to three case studies of deep excavations in sand where the subsidence was controlled by the deformation of the retaining structures finding a satisfactory and encouraging agreement.

## Introduction

**Figure 1:**
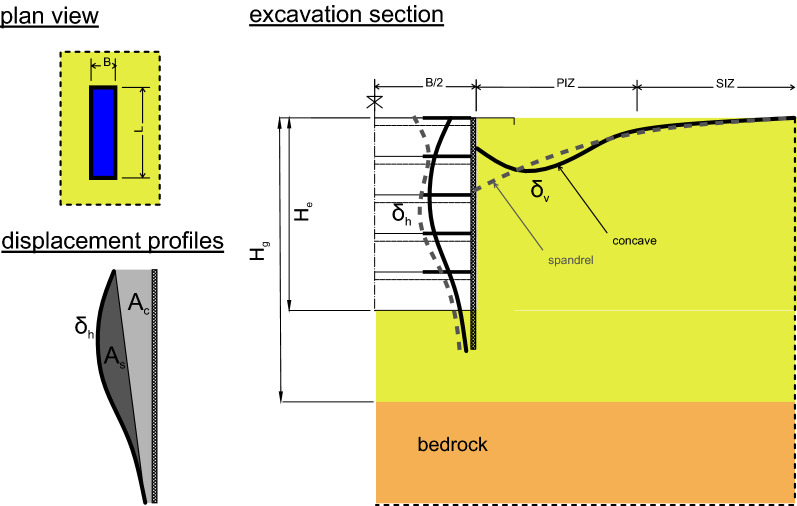
Sketch of the excavation with the main geometric parameters defining the problem.

The settlement around a deep excavation in urban environment are among the main construction issues to be addressed by the design because of the likely damages to nearby buildings and structures^1,2^. For the prediction of movements around excavations various proposals are available and rely on different approaches. The first and the simplest one, the semi-empirical approach, is based on the experience gained by interpreting data from case histories where displacement around excavation were measured and related to simple geometrical parameters such as the depth of the excavation ${H}_{e}$, the depth of the bedrock, ${H}_{g}$ if any, the size in plan as the length L or the width B (see Fig. 1).

Large databases on deep excavations case histories are available since relatively long time^3,4^ and proposed empirical methods have been tested at least partially on this extensive basis. Among the others reference can be made to the semi-empirical methods by Ou^5^, Clough and O’Rourke^6^ and Bowles^7^ for the transversal settlement trough (i.e., the settlement profile along the direction normal to the excavation edge) and to the method by Roboski and Finno^8^ for the longitudinal settlement trough (i.e., the settlement profile along a direction parallel to the excavation edge). Some more specific alternative has been proposed more recently for 2D transversal settlement trough in case of multipropped excavations in clay^9,10^. In the following section 2D empirical methods for the prediction of longitudinal and transversal settlement trough are first shortly reviewed. A combination of existing but slightly modified 2D methods was recently proposed by Russo et al.^11^ to analytically describe the full 3D subsidence trough around a rectangular deep shaft and is further developed in “A new closed form (shape) function describing the 3D subsidence trough” section.

The second approach, the numerical one, consists of the numerical prediction of the green field subsidence trough by means of a FEM or DFM model. Early and significant contributions on numerical analysis are those proposed by Clough and Hansen^12^, Finno and Harahap^13^, Whittle et al.^14^. As a matter of fact, a 2D simulation of the shaft excavation under plane strain assumption is currently a typical and nearly routinely design step^15^. Since the aim of the analysis is not only predicting bending moment and shear force acting on diaphragm wall but also the settlement trough behind the wall, extreme attention should be paid when selecting an appropriate constitutive soil model and simulating even apparently minor construction details^16^. L’Amante et al.^16^ for instance showed the importance of modelling the very initial stage of the construction of a reinforced concrete (i.e. r.c) diaphragm wall because of its large influence on both soil-structure interaction and on displacement field around the excavation compared to the simpler *wish-in-place* modelling option. The large computational resources available have recently pushed forward the frontier of numerical simulation capabilities allowing a relatively easy access to large 3D models. This has occurred more frequently with reference to the prediction of tunnelling settlement trough^17,18^ but contributions in the field of deep excavation are also available^19,20^. These latter contributions aimed mainly at describing 3D corner effects with reference to the horizontal displacement profile of the diaphragm walls.

In this paper 2D semi-empirical methods are reviewed and briefly compared in “Semi-empirical methods for 2D settlement predictions” section. Purposely selected methods are modified and combined to build a new 3D analytical description of the subsidence trough around a deep excavation in “A new closed form (shape) function describing the 3D subsidence trough” section. This description consists of a two variable subsidence function depending on several parameters; four independent parameters define the overall shape while the maximum settlement, usually occurring in the middle of the longer side L of the excavation, is a scale parameter.

In “3D FEM models to test the closed form expression” section 3D FEM nonlinear analyses of a deep excavation are presented and discussed. The subsoil layering is inspired by real soil profile typical of the urban area of Napoli (Italy) and the geometry of the excavation shaft is analogous to many metro-stations of the underground network developed in the last two decades. The influence of various factors as the props distribution, the depth of the excavation, the presence of the openings typically needed for the top-down construction procedure on the computed 3D settlement troughs are explored and presented. In “Computed FEM results and shape parameters” section the new two variable subsidence function is applied to describe the FEM results by optimised fitting and insights on the influence of the various above factors on the shape parameters required to setup the 3D closed form expression of the subsidence trough are finally achieved. The proposed closed form expression and some findings about the shape parameters are validated in “Validation of the closed form shape function: application to case studies” section via the application to three case studies of deep excavations in sand finally obtaining a satisfactorily and encouraging agreement.

## Semi-empirical methods for 2D settlement predictions

The deformation field around deep excavations is three-dimensional. The possibility of a simplified but reliable prediction by means of a two-dimensional model depends mainly on the two geometrical ratios $L/B$ (i.e., length on width) and $L/{H}_{e}$ (i.e., length on excavation depth). In the early excavation stages of an open pit (i.e., for large $L/{H}_{e}$ values) the 2D conditions are prevailing along nearly the whole side $L$ and the corner 3D effects are limited to a relatively small influence zone. The deeper the excavation the larger the influence zone around the corner with 3D effects getting more and more important on the whole displacement field^19,21,22^. Displacement fields around deep excavations have been studied in the past via 3D FEM analysis, but the research focussed on the horizontal displacement profile of the deformed supporting wall^19,20^. For the settlement prediction at ground surface simple 2D semi-empirical methods are more popular and more diffused in the engineering practice. For this reason, a brief review of semi-empirical methods for 2D settlement prediction around an excavation shaft is here proposed. The settlement field is generally considered strictly linked to the horizontal displacement profile of the retaining wall. The prediction of the surface settlement field is generally divided in two separate subproblems: i.e., the prediction of settlement troughs along two horizontal directions, respectively normal and parallel to the retaining wall. There are quite evident similarities between this methodology and the usual procedure adopted to manage the same issue with reference to a tunnel construction; the evolution of the 3D tunnelling excavation process is generally simply linked to the partial development of the transversal gaussian curve while the longitudinal settlement trough is considered less important and is simulated, if needed, with a cumulative error function. Similarly, in the case of open excavations research has paid more attention to the prediction of the settlement profile along the wall normal direction nearly neglecting the problem of settlement trough along the direction parallel to the wall. This probably occurred also because of the usually adopted assumption of analysing the excavation as a 2D plane strain problem. In the frame of this assumption the plane strain normal settlement trough is simply assumed to be invariable along the side itself. On the contrary the displacement field around a deep excavation is a full 3D problem.

With reference to the empirical prediction of the settlement trough in the direction normal to the wall the classical contribution by Peck^23^ or the subsequent proposals by Bowles^7^ and Clough and O’Rourke^6^ are still widely adopted in the preliminary design step of many deep excavation. However, all these works only suggested a conservative envelope of the expected settlement trough. Ou^5^ and Ou and Hsieh^24,25^ proposed on purely empirical bases two different shapes of the settlement trough using discontinuous functions and established a clear dependence of those shapes on the vertical profile of the diaphragm wall horizontal displacements. They divided the settlement trough into a *Primary Influence Zone* (PIZ) adjacent to the excavation, where large settlements occur, followed by a *Secondary Influence Zone* (SIZ) where settlements fade away. The SIZ and the PIZ were assumed with equal extension (see Fig. 1). Hence, they expressed the ground surface settlement ${\delta }_{v}$ normalised by its maximum value ${\delta }_{vmax}$ as a function of the PIZ extension and the distance $y$ from the excavation edge. In the original proposal by Ou^5^ the PIZ was the minimum between the excavation depth multiplied by two, i.e. $2\cdot {H}_{e}$, and the depth of the bedrock, ${H}_{g}$, if any. Furthermore, the shape of the settlement profile at the ground surface and normal to the embedded wall was assumed to be fully controlled by the horizontal displacement profile of the wall itself. To this aim Ou and Hsieh^25^ suggested to evaluate the ratio between the “cantilever area” ${A}_{c}$ and the difference ${A}_{s}$ between the total area and the cantilever portion on the lateral deflection profile of the wall (see Fig. 1): when it resulted that ${A}_{s}<1.6\cdot {A}_{c}$ a spandrel shaped settlement profile was expected while for ${A}_{s}>1.6\cdot {A}_{c}$ a concave shape was predicted.

The importance of the settlement profile in the direction parallel to the wall was first remarked by Roboski and Finno^8^. The authors^8^ used the complementary error function $f(x)=erfc(x)$ with its S-shape to describe a few in field observations. The only parameter to be fixed was named $A$ and represented the distance of the inflexion point of the settlement function from the corner of the excavation. A logarithmic function of the ratio $L/{H}_{e}$ calibrated on the limited basis of a couple of case histories of deep excavations in clay was proposed by the authors to predict at the design stage the value of the parameter $A$. As a general remark it is important to underline that 2D plane strain analysis might give inaccurate but conservative results in terms of settlement for the central section of an excavation wall. On the other hand, for sections near the corners the results of plane strain analysis would be much more inaccurate still leading to a conservative overestimate of the settlement amount but completely neglecting the potentially dangerous distortions induced by the 3D corner effects. For this reason, the 2D plane strain analysis can be considered neither accurate nor conservative.

## A new closed form (shape) function describing the 3D subsidence trough

Fast design tools to evaluate potential damage to the surrounding buildings and structures could be very useful at the preliminary design phases when, for instance, the exact location of the excavation has not yet been fixed. As concluded in the previous section such a tool should consider the 3D nature of the problem and the distortions close to the corner.

In the present section a simple semi-empirical equation is proposed for the description of the three-dimensional subsidence trough. This equation is the result of the combination of two separate proposals introduced first to describe the 2D settlement profiles along the normal and the parallel directions to the retaining wall.

The function $f(y)$ proposed to describe the settlement profile in the direction normal to the wall is:

1 $$\frac{{\delta }_{v}\left(y\right)}{{\delta }_{vmax}}=f\left(y\right)=\frac{a}{1+b\frac{y}{PIZ}+d{\left(\frac{y}{PIZ}\right)}^{2}}$$

where: ${\delta }_{vmax}$ and ${\delta }_{v}(y)$ are respectively the maximum settlement value and the settlement value at a distance $y$ from the embedded wall in the considered section while $a, b$ and $d$ are three fitting parameters. It is indeed simple to show that these three parameters are not independent of each other; as the value of the function $f(y)$ for $y\ge 0$ must lie in the range $\left[\mathrm{0,1}\right]$ a simple analytical relationship holds between the values of $a, b$ and $d$. The abscissa ${y}_{m}$ where the function reaches its maximum value is simply obtained by imposing that the first derivative of the function is equal to zero. This leads to the following relationships:

2 $$\frac{{y}_{m}}{\mathrm{PIZ}}=-\frac{1}{2}\frac{b}{d}$$

3 $$\mathrm{max}f{=f}_{m}=\frac{a}{1+\frac{b}{2}\cdot \left(-\frac{1}{2}\frac{b}{d}\right)}=\frac{a}{1+\frac{b}{2}\cdot \frac{{y}_{m}}{PIZ}}$$

The proposed function (1) describes the real settlement profile with limitation to positive values of the abscissa $y$; hence if expression (2) gives a positive value for ${y}_{m}$ then the predicted shape is concave and the maximum settlement occurs at ${y}_{m}$. On the other hand, if ${y}_{m}$ from expression (2) is negative obviously the restriction of $f\left(y\right)$ to the positive $y$ axis has a convex spandrel shape. In this second case the actual maximum of $f\left(y\right)$ coincides with the function value ${f}_{0}=a$ obtained at $y=0$:

4 $${\mathrm{max}{f}_{y\ge 0}=f}_{0}=a$$

As previously observed the maximum normalized settlement $\mathrm{max}{f}_{y\ge 0}$, must be equal to one and hence it results that for concave shape (i.e., if $-b/d>0$):

5 $${f}_{m}=\mathrm{max}f=1\Leftrightarrow a=1+\frac{b}{2}\cdot \left(-\frac{1}{2}\frac{b}{d}\right)=1+\frac{b}{2}\cdot \frac{{y}_{m}}{PIZ}$$

On the contrary for spandrel shape (i.e., if $-b/d\le 0$), it results:

6 $${f}_{0}=\mathrm{max}{f}_{y\ge 0}=1\Leftrightarrow a=1$$

and finally, both cases can be summarised as:

7 $$\max f_{{y \ge 0}} = 1 \Leftrightarrow a = \left\{ {\begin{array}{*{20}l} {1\because b/d \ge 0} \hfill \\ {1 + \frac{b}{2} \cdot \left( { - \frac{1}{2}\frac{b}{d}} \right) = 1 + \frac{b}{2} \cdot \left( {\frac{{y_{m} }}{{PIZ}}} \right)\because b/d < 0} \hfill \\ \end{array} } \right.$$

In conclusion, as anticipated above, the three parameters are not independent of each other and the value of $a$ is either 1 or depends on $b$ and $d$.

While parameter $a$ has a clear physical meaning in the process of the function definition, parameters $b$ and $d$ may be substituted by different parameters, obtained by some algebraic manipulation of the former ones, more directly linked to geometrical features of the profile which is very useful when the function is applied to empirical monitored profiles; in summary the chosen parameters are:

i. the function value at $y=0$: ${f}_{0}=f\left(y=0\right)=a$
ii. the derivative at $y=0$: ${f{^{\prime}}}_{0}=f{^{\prime}}\left(y=0\right)=-a\cdot b$
iii. the value of the function at $y=PIZ$:${f}_{1}=f\left(y=PIZ\right)=\frac{a}{1+b+d}$

After the above discussion is worth summarizing that in the case of a spandrel profile the shape function is completely defined by assigning the values of the two parameters ${f{^{\prime}}}_{0}$ (inclination of the settlement trough at the diaphragm wall) and ${f}_{1}$:

8 $${f}_{0}=1;{f{^{\prime}}}_{0}<0;0\le {f}_{1}<1\therefore \left\{\begin{array}{l}a={f}_{0}=1\\ {b=-f{^{\prime}}}_{0}\\ d=\frac{{f}_{0}}{{f}_{1}}+{f{^{\prime}}}_{0}-1\end{array}\right.$$

whilst for the concave profile the two parameters to be fixed are conveniently ${f}_{0}$ and ${f{^{\prime}}}_{0}$:

9 $${0<f}_{0}<1;{f{^{\prime}}}_{0}>0;{f}_{1}=\frac{{f}_{0}}{1-\frac{{{f}^{{^{\prime}}}}_{0}}{{f}_{0}}+\frac{1}{4}{\left(\frac{{{f}^{{^{\prime}}}}_{0}}{{f}_{0}}\right)}^{2}\frac{1}{1-{f}_{0}}}<1\therefore \left\{\begin{array}{l}a={f}_{0}\\ b=-\frac{{f}_{0}^{{^{\prime}}}}{{f}_{0}}\\ d=\frac{1}{4}{\left(\frac{{{f}^{{^{\prime}}}}_{0}}{{f}_{0}}\right)}^{2}\frac{1}{1-{f}_{0}}\end{array}\right.$$

**Figure 2:**
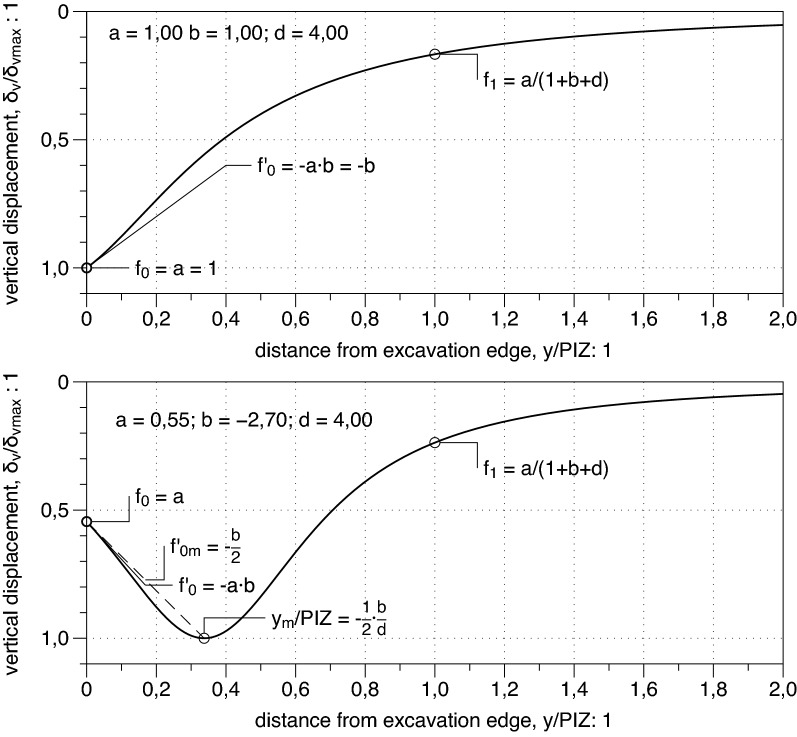
Settlement profiles in the direction normal to the wall as described by the new shape function.

An example of the proposed function (1) is plotted in Fig. 2 with reference to both concave and spandrel shapes. The relationships (8) and (9) are reported in the same figure and the meaning of the parameters defined in (i), (ii) and (iii) is graphically illustrated. Moreover, the significant relationship between the fitting parameter $b$ and the average slope ${f{^{\prime}}}_{0m}$ of function $f$ in the range between the wall and the abscissa of the maximum settlement (i.e., for $y\in \left[0,{y}_{m}\right]$):

10 $${f{^{\prime}}}_{0m}=\frac{{{f}_{m}-f}_{0}}{{y}_{m}/PIZ}=\frac{1-a}{{y}_{m}}PIZ=\frac{-\frac{b}{2}\cdot \frac{{y}_{m}}{PIZ}}{{y}_{m}}PIZ=-\frac{b}{2}$$

is also referred in Fig. 2.

**Figure 3:**
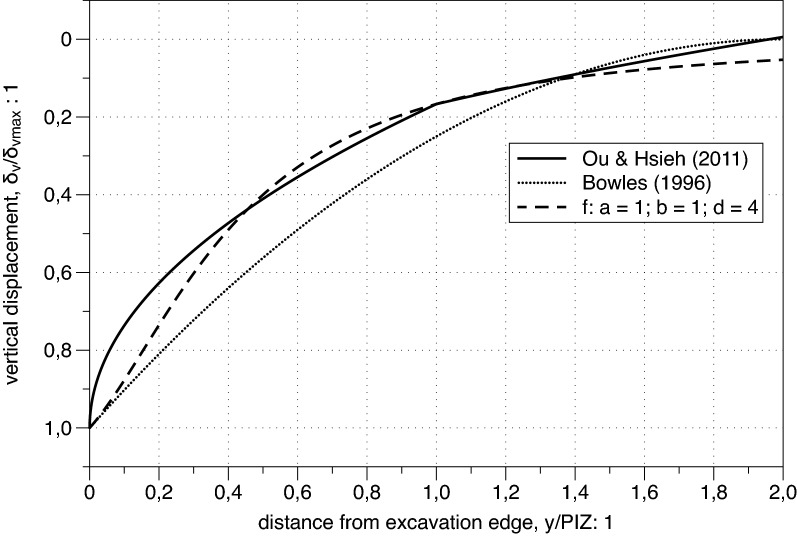
Spandrel settlement profiles.

**Figure 4:**
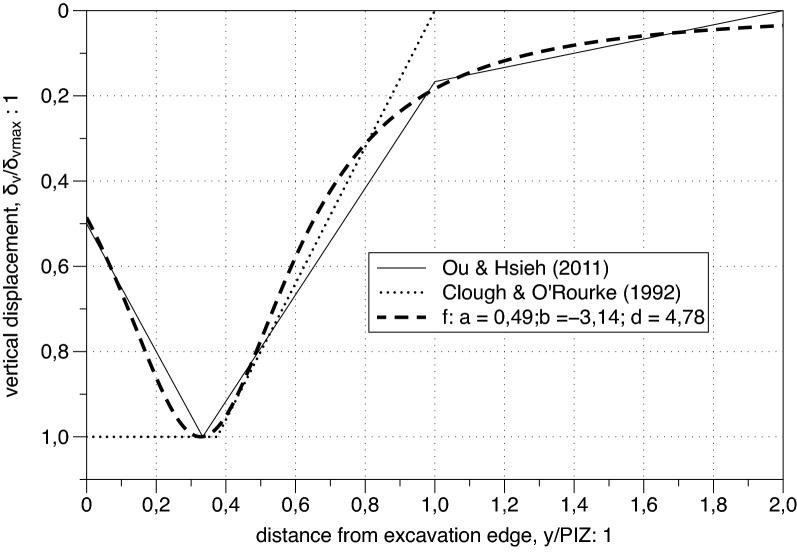
Concave settlement profiles.

In Fig. 3 the spandrel shape by Ou and Hsieh^25^ introduced in "Semi-empirical methods for 2D settlement predictions" is compared with the one suggested by Bowles^7^ and the new proposed profile arising from (1). An appropriate choice of the fitting parameters (e.g.: a = 1; b = 1; d = 4) in (1) leads to a profile intermediate between those of the two previous proposals (see Fig. 3) outlining the capabilities of the proposed function. Furthermore the rational form in (1) was selected because it allows to represent with a unique function also the concave settlement profile. In Fig. 4 (1) is capable of reproducing quite closely both the tri-linear discontinuous profile first proposed by Ou^5^ and one of the settlement envelopes proposed by Clough and O’Rourke^6^

It can be concluded that function (1) can be adapted to reproduce different settlement patterns depending on the value of the three parameters a, $b$ and $d$. Furthermore simple analytical conditions can be imposed on the more versatile function (1) to obtain significant similarities with previous proposed empirical settlement profiles. For instance, a sort of best fitting was obtained in the case of Fig. 4 adopting the following values of the parameters: $a=0.49; b=-3.14; d=4.78$. An obvious consequence is that the various calibration suggestions for the reviewed empirical methods could be easily adapted to the unique Eq. (1) for any transversal settlement pattern.

**Figure 5:**
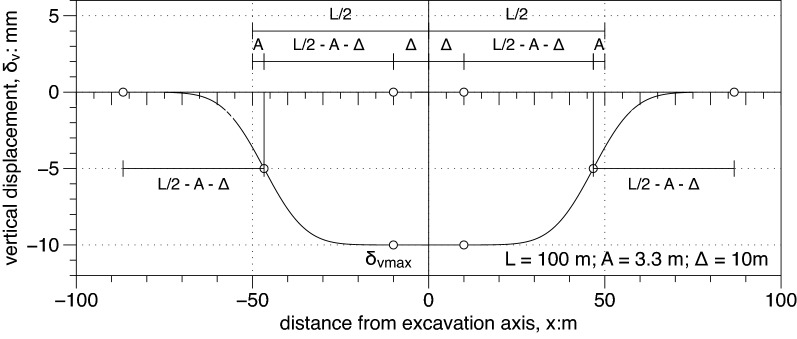
Settlement profiles in the direction parallel to the wall (Russo et al.^11^, $\Delta >0$)

**Figure 6:**
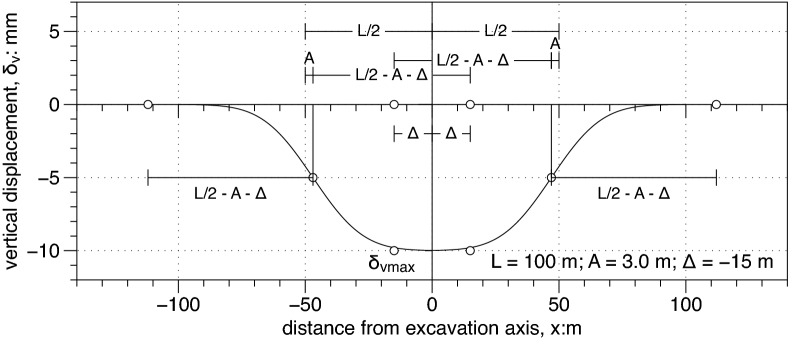
Settlement profiles in the direction parallel to the wall (Russo et al.^11^, $\Delta <0$)

In the direction parallel to the supporting wall a continuous function representing the settlement profile was recently proposed by Russo et al*.*^11^; it consisted in a modification of the function proposed by Roboski and Finno^8^. This function has the form:

11 $$\frac{{\delta }_{v}(x)}{{\delta }_{vmax}}=g\left(x\right)=\frac{1}{2}erfc\left[2.8\frac{\left|x\right|-\left(L/2-A\right)}{\left(L/2-A\right)-\Delta }\right]=\frac{1}{2}erfc\left[2.8\frac{\left|x/L\right|-\left(1/2-A/L\right)}{\left(1/2-A/L\right)-\Delta /L}\right]$$

with $x$ representing the distance from the center of the excavation and A the distance of the function inflection point from the corner of the excavation (see Figs. 5, 6). Russo et al.^11^ introduced the new coefficient $\Delta$ to simulate different longitudinal settlement profiles from very smooth (large $\Delta$) to very sharp ones (small and even negative $\Delta$). The closer to the plan strain condition in the middle of the side of the excavation the larger (and positive) the value of $\Delta$ .

By combining the two functions (Eqs. (1) and (11)) it is possible do define a function of two variables $x$ and $y$ representing the subsidence surface around an excavation. This combination is represented by the function:

12 $$\frac{{\delta _{v} \left( {x,y} \right)}}{{\delta _{{v\max }} }} = f\left( y \right) \cdot g\left( x \right) = \frac{1}{2}erfc\left[ {2.8\frac{{\left| {x/L} \right| - \left( {1/2 - A/L} \right)}}{{\left( {1/2 - A/L} \right) - \Delta /L}}} \right] \cdot \frac{a}{{1 + b\frac{y}{{PIZ}} + d\left( {\frac{y}{{PIZ}}} \right)^{2} }}$$

Equation (12) allows to define the overall settlement field around a deep excavation by applying it to both long and short sides of a rectangular excavation. Previously published charts based on 3D FEM analysis^5,26–28^ focused on 3D effects on horizontal displacement of the diaphragm walls indicating even a 40–50% reduction of the displacement close to the corner compared to the plain strain condition. However, if the focus is on settlements, the function ${\delta }_{v}\left(x,y\right)$ reported in (12) represents a new effective proposal to describe the 3D subsidence trough including the corner effects which affects mainly the parameters $A$ and $\Delta$. The overall function (12) depends on five parameters, four out of five being independent of the others.

## 3D FEM models to test the closed form expression

To test the (12) and to create a set of data to explore the variability of its independent parameters numerical 3D models were set up and solved in a FEM code (Plaxis 3D ver.20.1). The focus was on the influence on the shape of the 3D greenfield settlement trough of the geometry and of the excavation procedure with its apparently minor but typical optional construction details.

For empirical methods^28,29^ the calibration of the parameters is carried out by fitting data collected from case histories. On one hand these data are certainly very valuable but on the other hand they are often limited to readings performed along simple 2D alignment and furthermore they do not allow for the separate evaluation of the influence of various details. Settlement troughs obtained via numerical parametric analyses allow to look for 3D trends and to effectively separate the contribution of several different factors. In the present study the basic FEM model is inspired by several case histories of stations of the underground network in the urban area of Napoli^11,16,30–34^. It is worth recalling that the case history and the monitored behaviour were already successfully back-analysed by^35^ with a similar FEM model and also mentioning the fact that several station boxes have been constructed in recent years with similar geometry and procedure and many others will be in the close future.

**Figure 7:**
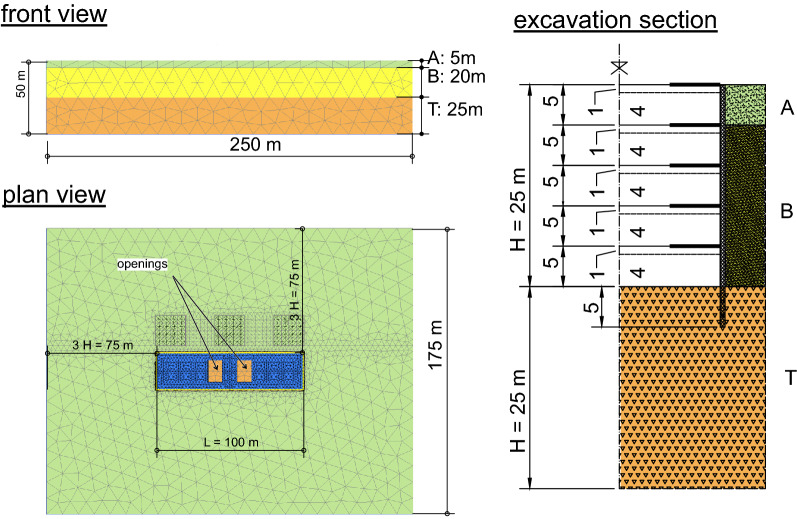
FEM model.

The deep shaft to be excavated has a rectangular plan with dimensions L × B = 100 m × 25 m and a depth H = 25 m. (see Fig. 7); the model had dimensions in plan of 250 m × 175 m, extended to a depth of 50 m and was composed by 80,840 10-nodes tetrahedral (4-stress point) elements with a total of 207,457 nodes.

In Fig. 7 the excavation section shows the geometry of the shaft with the props levels (thick black lines) and the subsequent intermediate positions of the excavation bottom (dashed lines).

The constant soil model adopted for the parametric analysis resound like a typical soil profile in the city of Napoli: a sequence of granular soil layers (soft to medium stiff sand) overlying a thick soft-rock stratum. The three layers sketched in the profile are: layer A, made ground; layer B, granular silty/sandy layer; layer T, soft rock bedrock (i.e., *Neapolitan Yellow Tuff*)^36,37^. The depth of the bedrock, ${H}_{g}$, was assumed to be 25 m which corresponds to the maximum excavation depth. The *Hardening Soil Small Strain Model*^38^ was selected to model the behaviour of layers A and B whilst the *Mohr–Coulomb Model* was optioned for the layer T. The physical and mechanical parameters (see Tables 1 and 2) were similar to those adopted for characterization of the true volcanic materials described in previous studies^17^. The FEM analyses were performed with the assumption of zero pore water pressure everywhere.

**Table 1:** Parameters for layers modelled with Hardening Soil Small Strain Model.

Layer	$\gamma$, kN m^−3^	$\varphi$, °	$c$, MPa	${G}_{0}$, MPa	${\gamma }_{0.7}$	${E}_{50}={E}_{oed}$, MPa	${E}_{ur}$, MPa	$\nu$
A	17	37	0	50	$1.30\times {10}^{-4}$	16	40	0.3
B	16	37	0	200	$2.63\times {10}^{-4}$	68	170	0.3

**Table 2:** Parameters for layers modelled with Mohr–Coulomb Model.

Layer	$\gamma$, kN m^−3^	$\varphi$, °	$c$, MPa	${\sigma }_{t}$, MPa	$E$, GPa	$\nu$
T	16	28	2.46	0.65	$2.60+0.78\times \left(z-{z}_{0}\right)$	0.3

### The excavation procedure

A classical *top-down* procedure was adopted for the shaft construction with intermediate rc slabs installed (cast in place) on the excavation temporary bottom. The diaphragm walls were modelled as *wish in place*. For this reason the computed displacements do not include movements generated by the excavation and the subsequent construction stage of the r.c. diaphragm walls. L’Amante et al.^16^ provided simple tools to evaluate this type of movements. However, two different construction processes were analysed. In the former one, indicated in the following as *Top*-*Propped case* (TP), the first prop was installed just after a preliminary, very shallow, excavation (1 m below the prop axis). In the latter case, called *Top-Unpropped case* (TU), the first prop was installed after a deeper excavation (down to a depth of 6 m below original ground surface); this latter procedure was in fact used in many sites in Napoli and in other historical cities to allow for the mandatory preliminary archaeological checks. The properties of the anisotropic plates adopted for modelling the r.c. diaphragm walls are in Table 3. The intermediate slabs were modelled as isotropic linear elastic plate (axial stiffness per unit length $EA=6.3 \; \text{GN}/\text{m}$). In the central part of each slab two openings 10 m × 15 m are sketched in Fig. 7. These openings are generally needed for soil removal but may have different locations and sizes. To evaluate the influence of the openings the analyses were repeated also without the openings. Thus, as summarized in Table 4, four cases were analysed in greenfield conditions.

**Table 3:** Properties of plate elements adopted for r.c. diaphragm walls (v vertical direction; h horizontal direction contained in plate plan; n horizontal direction normal to plate plane).

Thickness, m	${E}_{v}$, GPa	${\nu }_{vh}$	${E}_{h}/{E}_{v}$	${G}_{vn}$, GPa	${G}_{vh}={G}_{hn}$, GPa
0.60	31.4	0	0.1	15.7	1.57

**Table 4:** Analysed cases.

Field condition	Propping scheme	Openings	ID	
Greenfield	Top propped	Yes	G	TP
	Top unpropped	Yes		TU
	Top propped	No		TPNO
	Top unpropped	No		TUNO
Buildings	Top propped	Yes	B	TP
	Top unpropped	Yes		TU

## Computed FEM results and shape parameters

**Figure 8:**
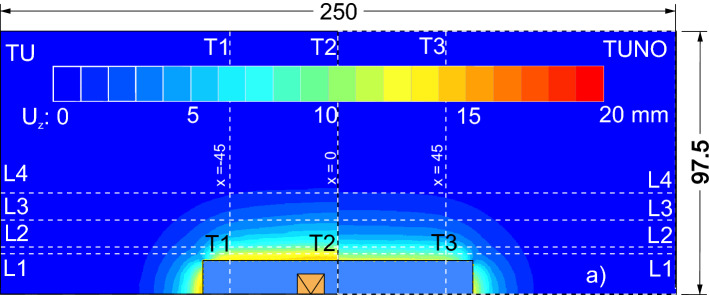
Settlement around the excavation, case TU and TUNO.

**Figure 9:**
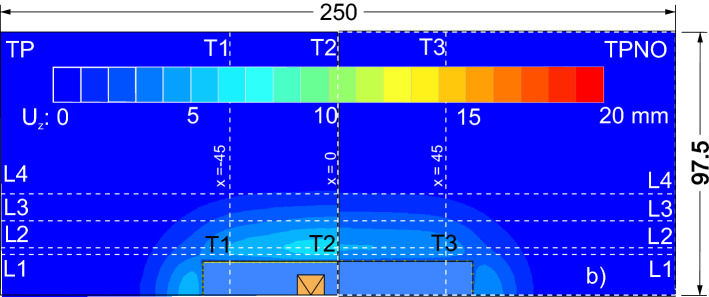
Settlement around the excavation, case TP and TPNO.

A plan view of the computed settlement at the ground surface is reported with coloured contour maps in Figs. 8 and 9 for the four analysed cases (i.e., TP, TU, TPNO and TUNO); it is clearly appreciable the three-dimensional nature of the deformative phenomenon that develops around the excavation. Figures 8 and 9 show that both the investigated details of the excavation affect the subsidence troughs. In the unpropped cases (ie. TU and TUNO) the settlements are larger than in the propped cases (i.e., TP and TPNO) and similarly the cases with the openings show larger settlement compared to the cases without openings. The different shapes of the 3D settlement troughs will be discussed in the following sections. To this aim a best fitting exercise of the 3D computed settlement field at the ground surface is presented in the “3D settlement trough” section using Eq. (12) while in the following two sections a separate discussion of the computed transversal and longitudinal profiles is first carried out.

### Settlement transversal profiles

**Figure 10:**
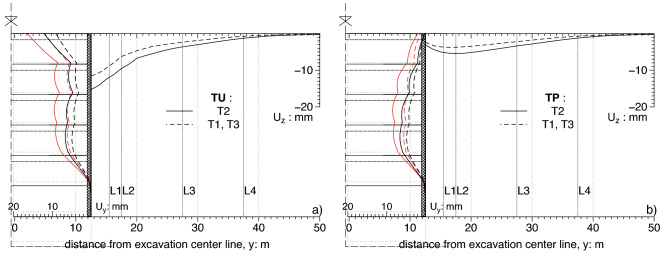
Displacements along sections normal to the wall: (**a**) analyses TU; (**b**) analyses TP.

A plot of the results along the transversal sections is reported in Fig. 10. The vertical component (Uz) of ground surface displacements and the horizontal component (Uy) of the diaphragm wall displacements along the transversal section T1,T3 and T2 are plotted in Fig. 10 for the cases TU and the case TP.

In the case TU the ratio ${A}_{s}/{A}_{c}$ for the two sections $x=0 \; \text{m}$ (Section T2) and $x=\pm 45 \; \text{m}$ (Section T1 and T3) was computed as 1.56 and 1.52 respectively; these values are below the threshold of 1.6 fixed by Ou^5^ and confirm the validity of this ratio as a predictor of a spandrel shape of the transversal 2D settlement trough at the ground surface. Also in the case TP (Fig. 10) the concave shapes of the profiles are coherent with the value of the computed ratio ${A}_{s}/{A}_{c}$ = 43, well above the mentioned threshold. On the other hand, it must be recognized that both the spandrel and the concave shapes do not follow the profiles (i.e. location of the maximum displacement, maximum extension of the profile) predicted by the empirical method proposed by by Ou and Hsieh^25^. These profiles clearly depend on the different excavation procedures and on the geometry of the excavation pit which have no role in the above method. The influence of the openings on the transversal profile is nearly negligible and is not discussed in this section; it will be considered in the 3D section (“3D settlement trough”).

### Settlement longitudinal profiles

**Figure 11:**
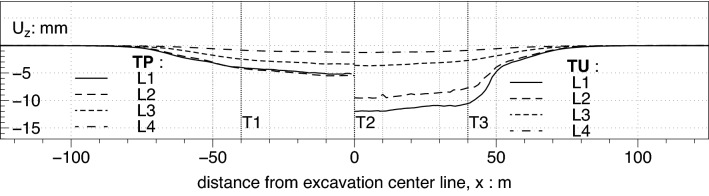
Settlement along sections parallel to the wall: TU (right) and TP (left).

A representation of the results along various longitudinal sections (from L1 up to L4) is reported in Fig. 11. The proposed comparison is between propped, TP, and unpropped, TU case The general shape of the profile and the relative extension of the central zone, where the concavity is upwards is controlled by the two parameters $A$ and $\Delta$ introduced by Russo et al.^11^ in the definition of the function $g(x)$. Here it is just worth noting that the unpropped case show larger settlement in the central zone of the long side while the extension of the central “plateau” zone is larger in the propped case while the parameters A and Δ will be determined in the following section by fitting the overall subsidence trough with the Eq. (12).

### 3D settlement trough

The settlement data resulting from the 3D FEM analyses were fitted using the function (12) (it was used a Matlab R2020 script based on the Curve Fitting Toolbox 3.5.11); the function is completely defined by four independent shape parameters $\left(b,d,A,\Delta \right)$ plus the maximum settlement ${\delta }_{vmax}$ as scale factor.

**Figure 12:**
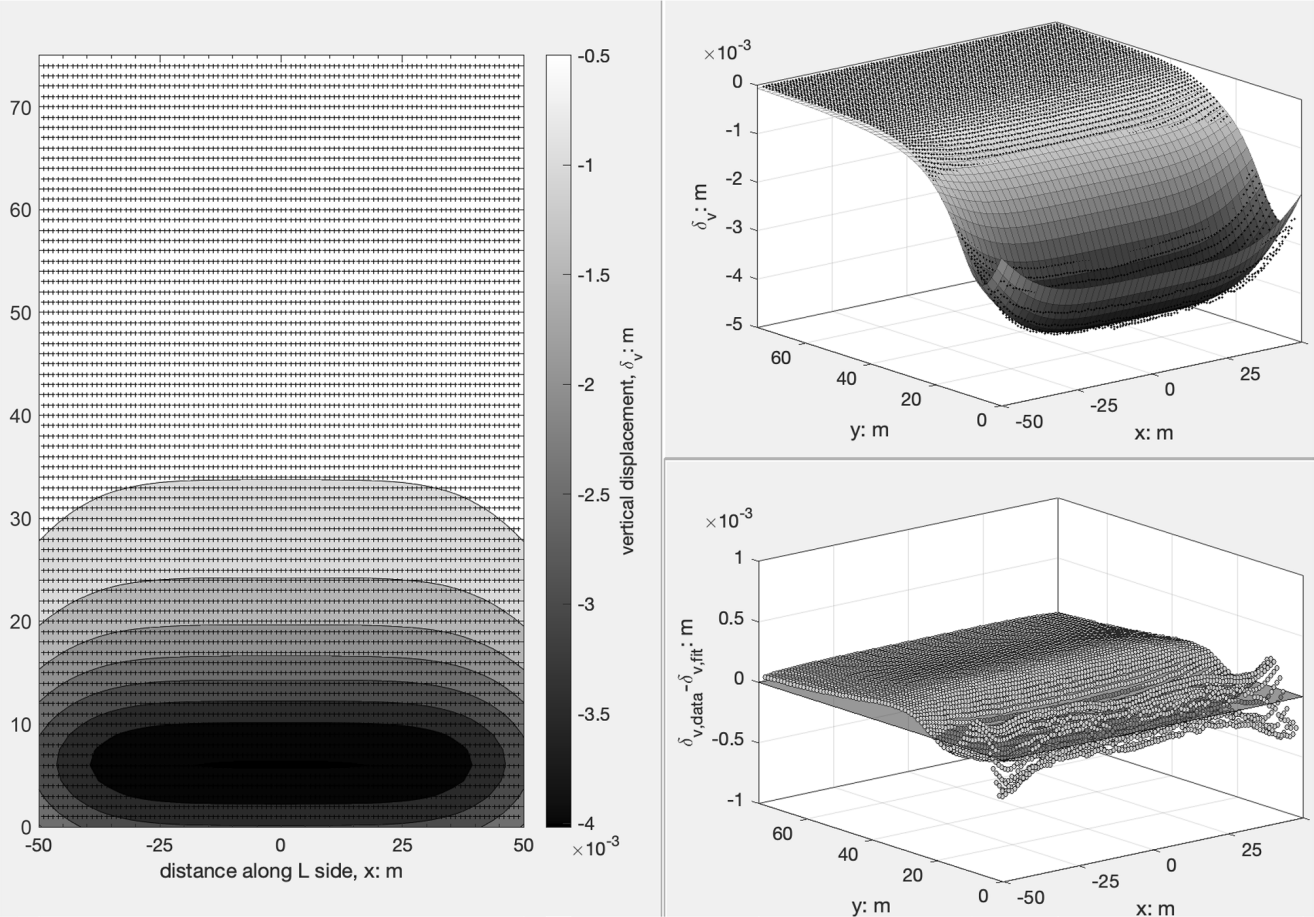
Fitted surface, comparison between fitted and computed settlement and residuals distribution (case TPNO).

The fitted surface obtained for the case TPNO is plotted as an example on the long side $L$ of the excavations in Fig. 12; on left side a grey-scale colour map diagram is plotted, on the right side (top) a straightforward comparison between the fitting surface and the computed data is reported while on the right bottom the residuals are represented. The quality of the fitting is very satisfactory (see Fig. 12) because: (i) ${R}^{2}$ value is very close to 1; (ii) the absolute values of residuals is of the order of a few hundredths compared to the fitted settlement; (iii) the residuals spatial distribution do not show any hidden trend to be further investigated. Similar comments apply to the fitting results of both sides of the excavations and for all the computed cases even if the fitting parameters are obviously different. The values of the fitting parameters and of the coefficient of determination ${R}^{2}$ are summarized for all the cases analysed via FEM in Table 5. They are referred to both sides of the excavation and to five different bottom excavation depths representing ten different cases in terms of the geometrical ratio $L/{H}_{e}$. In the following two subsections, suggestions on how to fix the values of the six parameters ${f}_{0},{f{^{\prime}}}_{0},{f}_{1},{y}_{m}$, $A$ and $\Delta$, as a function of the excavation procedure and of the geometrical ratios of the excavation shaft are reported. For the transversal part of the profile, it is worth recalling that $b$ and $d$ are preferred as fitting parameters but ${f}_{0},{f{^{\prime}}}_{0},{f}_{1},{y}_{m}$, are here preferred because have a clear geometrical meaning and in “A new closed form (shape) function describing the 3D subsidence trough” section analytical relationships among them are investigated and established.

**Table 5:** Fitting results.

Side	ID	Top prop	Openings	${H}_{e}$(m)	$PIZ$(m)	${R}^{2}$	${\delta }_{vmax}$(mm)	$\frac{A}{L}or\frac{A}{B}$	$\frac{\Delta }{L}or\frac{\Delta }{B}$	$b$	$d$
L	TP	Yes	Yes	6	12	0.986	0.552	− 0.042	0.211	− 0.115	0.828
				11	22	0.992	1.678	− 0.093	− 0.260	− 1.583	4.031
				16	25	0.991	3.351	− 0.060	− 0.412	− 2.214	4.864
				21	25	0.987	4.734	− 0.050	− 0.516	− 2.189	4.344
				25	25	0.987	4.978	− 0.048	− 0.593	− 2.179	4.294
	TPNO	Yes	No	6	12	0.986	0.542	− 0.032	0.252	− 0.129	0.818
				11	22	0.992	1.515	− 0.036	0.162	− 1.531	3.859
				16	25	0.991	2.870	− 0.043	0.057	− 2.142	4.750
				21	25	0.987	3.922	− 0.055	− 0.009	− 2.129	4.306
				25	25	0.987	4.121	− 0.067	− 0.103	− 2.126	4.242
	TU	No	Yes	6	12	0.996	7.578	0.023	0.324	− 2.309	15.802
				11	22	0.998	9.217	0.018	0.285	− 0.545	24.516
				16	25	0.995	10.960	0.009	0.191	1.069	14.810
				21	25	0.991	11.967	− 0.005	0.048	0.995	9.421
				25	25	0.990	12.114	− 0.010	0.008	0.954	9.052
	TUNO	No	No	6	12	0.996	7.578	0.023	0.324	− 2.309	15.802
				11	22	0.999	9.163	0.018	0.300	− 0.494	25.133
				16	25	0.997	10.812	0.011	0.257	1.274	15.553
				21	25	0.993	11.627	0.004	0.214	1.293	10.032
				25	25	0.993	11.687	0.003	0.212	1.268	9.747
B	TP	Yes	Yes	6	12	0.995	0.705	− 0.049	− 0.171	− 0.356	1.530
				11	22	0.997	1.846	− 0.063	− 0.341	− 2.116	5.957
				16	25	0.994	3.117	− 0.097	− 0.464	− 2.451	6.318
				21	25	0.989	4.066	− 0.133	− 0.525	− 2.283	5.257
				25	25	0.989	4.205	− 0.140	− 0.530	− 2.254	5.111
	TPNO	Yes	No	6	12	0.995	0.694	− 0.049	− 0.174	− 0.367	1.515
				11	22	0.997	1.816	− 0.061	− 0.345	− 2.120	5.918
				16	25	0.994	3.063	− 0.096	− 0.469	− 2.471	6.321
				21	25	0.990	3.989	− 0.132	− 0.531	− 2.307	5.281
				25	25	0.989	4.125	− 0.139	− 0.537	− 2.277	5.136
	TU	No	Yes	6	12	0.996	7.803	0.089	− 0.152	− 2.253	15.553
				11	22	0.998	9.385	0.068	− 0.213	− 0.998	25.207
				16	25	0.996	10.732	0.045	− 0.264	0.398	17.618
				21	25	0.992	11.510	0.021	− 0.305	0.744	11.476
				25	25	0.991	11.600	0.017	− 0.310	0.757	10.822
	TUNO	No	No	6	12	0.996	7.803	0.089	− 0.152	− 2.253	15.553
				11	22	0.998	9.342	0.069	− 0.212	− 1.035	25.413
				16	25	0.996	10.648	0.046	− 0.264	0.358	17.887
				21	25	0.992	11.404	0.023	− 0.304	0.716	11.682
				25	25	0.991	11.486	0.019	− 0.309	0.727	11.037

#### Parameters of $f\left(y\right)$ profile 

**Figure 13:**
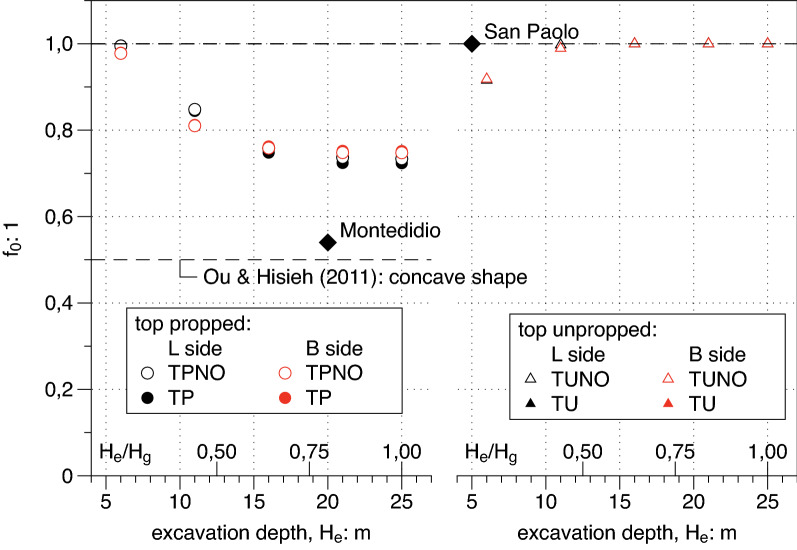
Dependency on the excavation depth H_e_ of the value ${\mathrm{f}}_{0}$ of the transversal settlement function $\mathrm{f}$ at the edge of the excavation.

**Figure 14:**
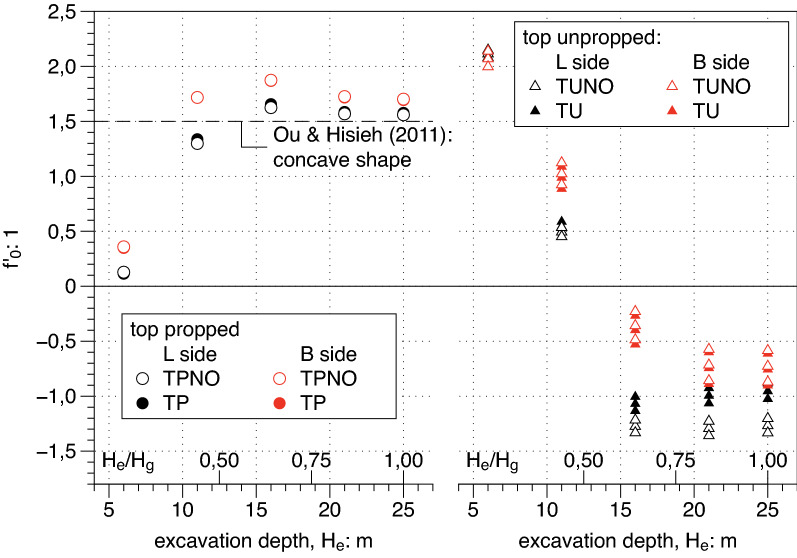
Dependency on the excavation depth H_e_ of the slope ${\mathrm{f{^{\prime}}}}_{0}$ of the transversal settlement profile path at the edge of the excavation.

**Figure 15:**
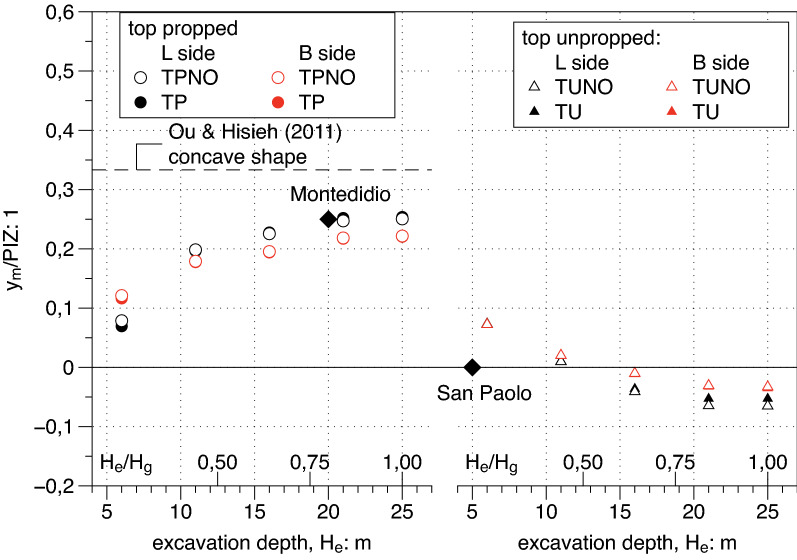
Dependency on the excavation depth H_e_ of the normalised distance of the maximum settlement location ${\mathrm{y}}_{\mathrm{m}}/\mathrm{PIZ}$ from the edge of the excavation.

In Figs. 13, 14 and 15 the three parameters ${f}_{0},{f{^{\prime}}}_{0},{y}_{m}$ are plotted versus the depth of the excavation ${H}_{e}$. The plots have two side by side windows referred to propped (left side) and unpropped (right side) cases, while red symbols are referred to the short side $B$ and black symbols are referred to the long side $L$.

It is clearly shown by the right side of the three plots that for unpropped cases the settlement transversal profile has a spandrel shape with the only exception of the very unrealistic case characterized by the shallowest depth (i.e. ${H}_{e}= 6\; \text{m}$) where the profile is concave having at the same time ${f}_{0}<1$, ${f{^{\prime}}}_{0}>0$ and finally ${y}_{m }/PIZ>0$. For propped cases (i.e. TP cases on left side) all available data confirm the occurrence of concave shapes even if with different values and a clear trend: the deeper the excavation ${H}_{e}$ the more pronounced the concave shape. In the same plots the suggested values by Ou and Hsieh^25^, are reported for comparisons. A good match is found between the computed results and the suggested value for the parameter ${f{^{\prime}}}_{0}$ which represent the inclination of the settlement transversal trough at the diaphragm wall. The same agreement is not confirmed for the other two plotted parameters ${f}_{0},{y}_{m}$. which respectively show a decreasing and an increasing trend with the excavation depth ${H}_{e}$ differently from the suggested constant value by Ou and Hsieh^25^. Both these dependencies are of purely geometrical nature and should be taken into account in the definition of function $f\left(y\right)$ (1) .

**Figure 16:**
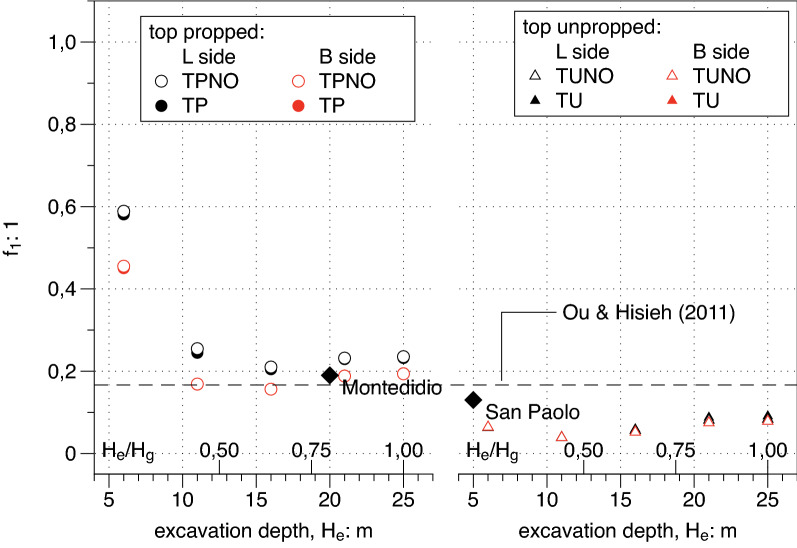
Dependency on the excavation depth H_e_ of the value ${\mathrm{f}}_{1}$ of the transversal settlement function $\mathrm{f}$ at a distance $\frac{{\mathrm{y}}_{\mathrm{m}}}{\mathrm{PIZ}}=1$.

To complete the description of the transversal profile in Fig. 16 the value of the parameter ${f}_{1}$ as deduced by fitting is plotted versus the excavation depth ${H}_{e}$_._ Unpropped cases show a constant value which is smaller than the value suggested by Ou and Hsieh^25^ while propped cases (left side of the plot) show a very good agreement with suggested values with the only exception of the very unrealistic case corresponding to ${H}_{e}=6\; \mathrm{ m}$. Thus, on this parameter the suggestion by Ou and Hsieh^25^ is either confirmed (for propped cases) or at least conservative (for unpropped cases).

**Figure 17:**
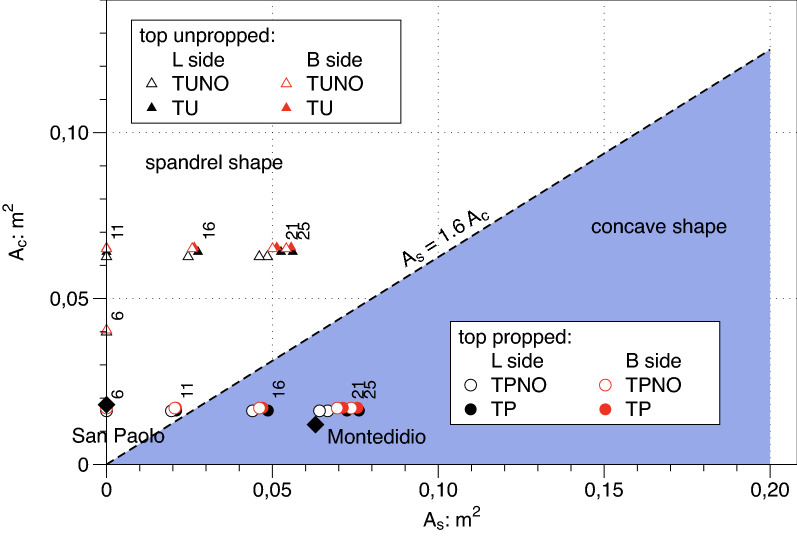
Areas of the horizontal displacement profiles of the embedded wall from FEM results: ${\mathrm{A}}_{\mathrm{c}}$ area of the cantilever portion of the displacement profile; ${\mathrm{A}}_{\mathrm{s}}$ difference between the total area and the cantilever portion.

The values of the two areas ${A}_{c}$ and ${A}_{s}$, as defined in “Semi-empirical methods for 2D settlement predictions” section and calculated from the FEM results, are finally reported in Fig. 17. This diagram permits to complete the discussion on $f\left(y\right)$ function. A similar diagram was proposed on empirical basis by Ou and Hsieh^25^. According to the discussed results it can be simply concluded that the validity of the predictor is here confirmed: all the propped cases (i.e. TP) have a pronounced concave shape with the only exception of the case ${H}_{e}=6 \; \text{m}$ which falls close but just outside the purple area; for the unpropped cases the spandrel shape is confirmed with the only exception of the case ${H}_{e}=6 \; \text{m}$ which was however considered as highly unrealistic.

#### Parameters of the profile g(x) 

**Figure 18:**
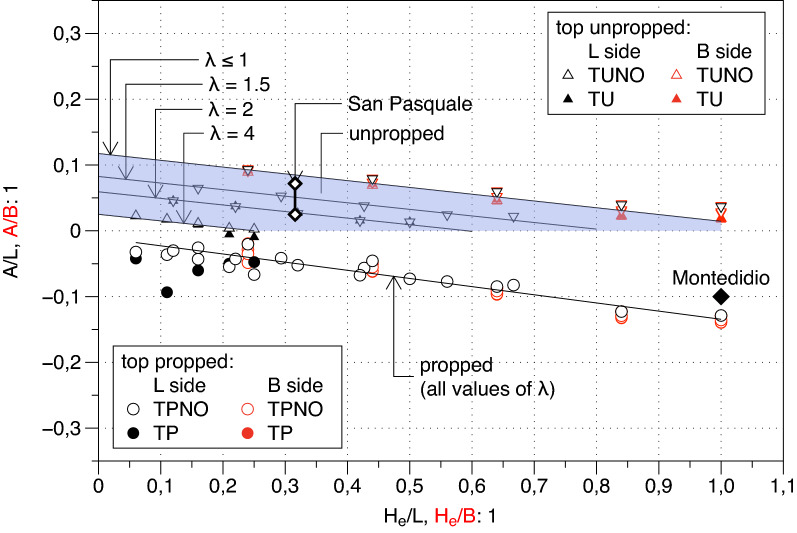
Normalised distance of the inflexion point from the corner of the excavation, A/L.

**Figure 19:**
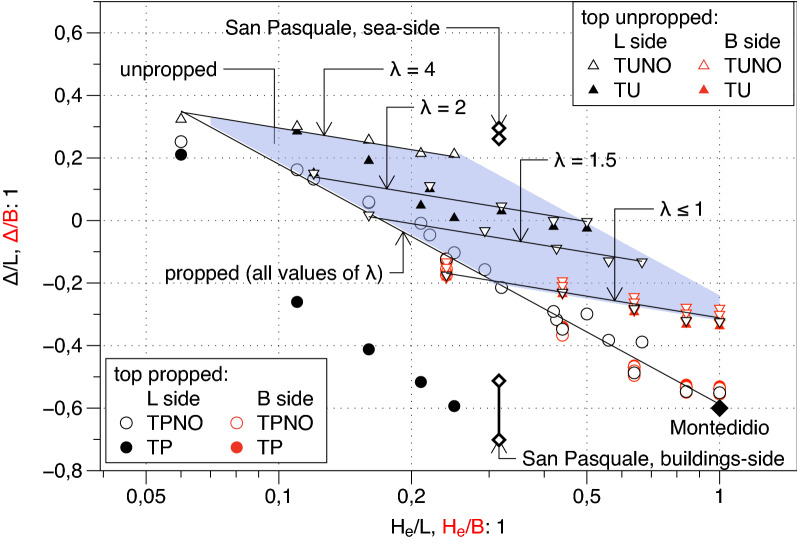
Normalised plateau length Δ/L (Δ/B).

In Figs. 18 and 19 the two fitting parameters $A$ and $\Delta$ normalised with reference to the length of the pertaining excavation side (i.e., $L$ or $B$) are plotted versus the depth of the excavation ${H}_{e}$ normalised by the same length.

Roboski and Finno^8^ for an $L$ wide excavation proposed the following empirical relationship between the parameter $A$ and the excavation depth ${H}_{e}$:

13 $$2A/L=-0.069\cdot \mathrm{ln}\left({H}_{e}/L\right)-0.03$$

It was based on the measured performance of a couple of excavations in clayey subsoils. The trend of the parameter A in Fig. 18 does not agree with the general function (13). In the case of propped excavations, the results show a clear simple and linear dependence of the distance $A$ on (i) the excavation depth ${H}_{e}$ with a small slope and a nearly 0 intercept ${I}_{A}$ as summarized in the relationship reported in the Table 6 which is valid no matter about the presence of the openings. On the contrary for the Unpropped cases (the triangles in Fig. 18) whilst the slope remains constant the exact value of the intercept ${I}_{A}$ depends on the excavation side lengths ratio $\lambda$ ($={L}_{p}/{L}_{t}$; i.e. the ratio between the length ${L}_{p}$ of the side of the excavation parallel to the settlement trough and the length of the transversal side ${L}_{t}$) that is equal to 4 for side $L$ and 0.25 for side $B$. However, as shown by the Fig. 18, in all considered cases the value of $A$ fall in the narrow range [0–0.1]. It is to underline that to fully support such a concluding remark some further cases with different values of $\lambda$ (i.e., $\lambda$ = 2.0, 1.5, 1.0, 0.67 and 0.5) were analysed in addition to the fundamental ones listed in Table 4 and the corresponding $A$ values are plotted in Fig. 18 by means of inverted triangle. In Table 6 a summary of the finding for the parameter A for both Unpropped and Propped cases is reported.

**Table 6:** Relationship between A, Δ and the excavation depth ${\mathrm{H}}_{\mathrm{e}}$.

Cases	$A/{L}_{p}$ and $\Delta /{L}_{p}$	Note
Unpropped	$A/L_{p} = - 0.1 \cdot \left( {H_{e} /L} \right) + I_{A} \Delta /L_{p} = - 0.1 \cdot \ln \left( {H_{e} /L} \right) + I_{\Delta }$	${I}_{A}={I}_{A}\left(\lambda \right)$; $A/{L}_{p}\in [0, 0.1]$ ${I}_{\Delta }={I}_{\Delta }\left(\lambda \right)$
Propped	$A/L_{p} = - 0.1 \cdot \left( {H_{e} /L} \right) - 0.02\Delta /L_{p} = - \frac{1}{3} \cdot \ln \left( {H_{e} /L} \right) - 0.59$	

As regards the parameter $\Delta$ a graphical representation similar to that just commented for the parameter $A$ is reported in Fig. 19. The parameter Δ results to be a decreasing function of the excavation depth analogously to the parameter $A$. It is also clearly shown that the presence of the openings affects the relationship in both Propped and Unpropped Cases. Similarly, to what observed for the parameter $A$ in the Unpropped cases only, the side lengths ratio $\lambda$ affects the relationship between $\Delta$ and the excavation depth ${H}_{e}$ . This relationship results to be linear in a semi-log plane but both the slope and the intercept change in a relatively wide range. On the contrary for the Propped cases the log-linear relationship between the parameter $\Delta$ and the excavation depth is unique (not depending on the ratio *λ*).

In this case the presence of the opening on the long side L play a very important role (full circles cases). In such a case the decreasing trend of Δ with the excavation depth is confirmed but the relationship is significantly different from the general equation found above. In Table 6 a summary of the finding for the parameter Δ for both Unpropped and Propped cases is reported. Furthermore, it can simply be noted that also for unpropped case the openings induce a reduction of the parameter Δ compared to the case without openings (i.e., TU compared to TUNO). This is a somewhat expected result because the presence of the openings is responsible for a sharper transition across the symmetry axis compared to the case without the openings.

## Validation of the closed form shape function: application to case studies

Three case studies of excavations in sand carried out in the city of Napoli are here presented to be used in the validation of the functions $f\left(y\right)$ and $g\left(x\right)$ and their combination with parameters calibrated on the FEM results application.

The first one is the case of *Montedidio* underground car park built with top-down procedure in the historical city center. The second one is referred to another underground car park build in the *Fuorigrotta* borough for the *San Paolo* mall. The third one is referred to an underground metro station excavated in the fashion district of *Chiaia*. In all the cases the excavations were carried out in sand overlying a bedrock. In the first two cases the groundwater table was deeper than the excavation depth while for the third case the low permeability of the bedrock and the presence of the sea very close to the excavation inhibited the groundwater lowering outside the excavation. Hence the settlements outside the excavation shaft were in all the cases governed by the retaining system deformations.

### *Montedidio* project

A multi-storey underground parking was built in a site very close to existing masonry buildings in the historical centre of the city of Napoli at the top of the *Montediddio* hill. The excavation reached a maximum depth ${H}_{e}$ = 20 m, about 11.5 m of it was dug in pyroclastic sands and the remaining in the Neapolitan yellow tuff bedrock. The walls of the excavation and the pillars that support the floors are made of tam micro-piles. The excavation was carried out with the top-down technique. The slabs acted as stiff struts and temporary ground anchors were also adopted to increase the stiffness and reduce the displacement. Horizontal displacements with electrolevels tilt sensors, subsidence of buildings very close to the edge of the excavation and loads in ground anchors with vibrating wire load cells were monitored during construction. More details on the case can be found in Russo and Viggiani^39^.

**Figure 20:**
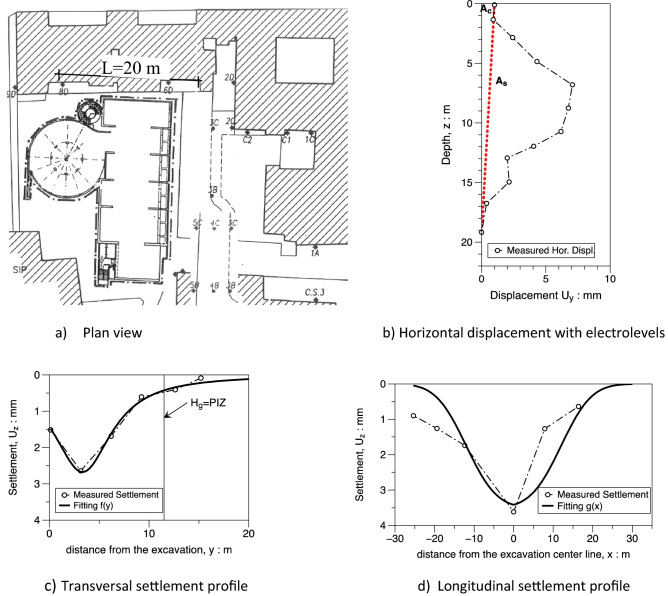
Plan view of the Montedidio site with Horizontal displacement and Settlement: comparison between predicted and measured behaviour (after Russo and Viggiani^39^).

A plan view of the site is shown in Fig. 20a; the dimension L = 20 m of the excavation shaft is reported and benchmarks installed for topographic survey are represented. The horizontal displacement measured at the end of the excavation process via electrolevels tilt sensors is plotted in Fig. 20b; it results that the ratio As/Ac = 5,2 is well above 1.6 and “predict” a concave transversal shape. The transversal settlement profile in Fig. 20c has a concave shape which is fitted using the $f\left(y\right)$ function (1). The value of ${f}_{0}$=0.54 is in the range between 0.5 and 0.7 respectively suggested by Ou and Hsieh^25^ and obtained by FEM computations presented in the paper (see Fig. 13). The same occurs for the parameter $\frac{{y}_{m}}{PIZ}=$ 0.25 that falls in the range 0.20–0.33 (see Fig. 15) and for the parameter ${f}_{1}=$ 0.19 in the range 0.16–0.20 (see Fig. 16). Finally, the values of $A$ = − 2 m and $\Delta$ = − 12 m can be calculated applying the relationships in Table 6. The function $g\left(x\right)$ of the Eq. (11) produces the satisfactory fitting of the measured longitudinal settlement profile of Fig. 20d). However it has to be recognized that the measured performance is certainly affected by the building stiffness in this case.

### *San Paolo* mall project 

A multi-storey underground parking was built during the construction of a shopping center in a site very close to existing reinforced concrete frame buildings in the *Fuorigrotta* borough in the western area of the city of Napoli. The maximum depth of the excavation is variable and ranges from 5 to 18 m and it was entirely carried out in sand above the groundwater table. The perimetral walls were made by reinforced concrete diaphragm walls. The excavation of the deeper areas was carried out using ground anchors as temporary support. Horizontal displacements measured with inclinometric probe and settlement either of the soil and of the surrounding buildings were monitored during the construction. More details on the case can be found in Fenelli and Ramondini^40^ and Fenelli and Pagano^41^.

**Figure 21:**
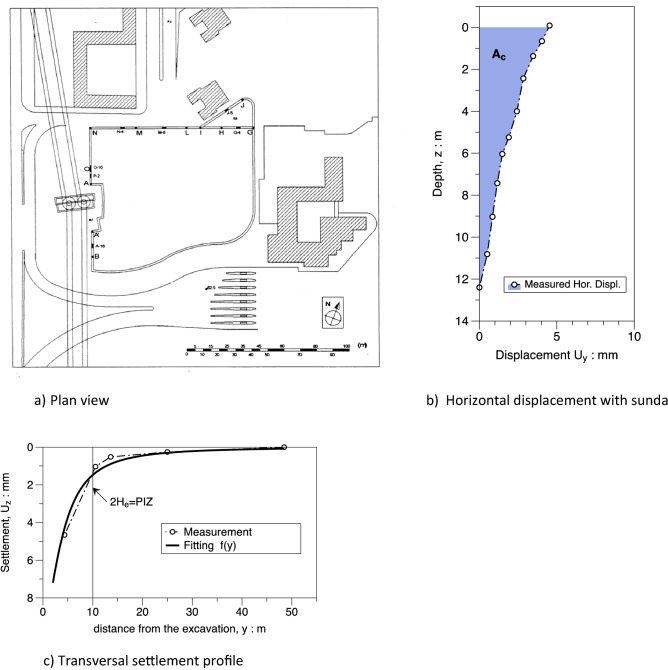
Plan view of the San Paolo mall site with Horizontal displacement and Settlement: comparison between predicted and measured behaviour (after Fenelli and Ramondini^40^).

A plan view of the site is shown in Fig. 21a; the excavation shaft is plotted and the panels of the diaphragm wall monitored via inclinometric probe are represented. The horizontal displacements measured at the end of the excavation process with reference to the panel J4 (H_e_ = 5 m) are reported in Fig. 21b showing a cantilever shape as usual for an unpropped excavation wall. Finally the settlement transversal profile measured behind the panel J4 and successfully fitted with $f\left(y\right)$ (Eq. 1) is represented in Fig. 21c. In this case the values obtained by fitting are ${f}_{0}$ = 1, ${f}_{1}$ = 0.13 which are in good agreement with the range of values obtained by FEM and plotted in Figs. 13 and 16 respectively.

### *San Pasquale* station project 

A 27 m deep excavation in a pyroclastic sand was constructed in the urban area of Napoli (Italy) to be used as station named *San Pasquale* along the downtown stretch of line 6 close to the sea. The main excavation is supported by T-section reinforced concrete (RC) diaphragm walls. The presence of valuable historical edifices on one side of the excavations suggested the selection of a top-down construction procedure. The reinforced concrete intermediate slabs were used as permanent struts and two large central openings were needed to allow for soil removal during the excavation stages. Full settlement troughs were observed along two different alignments. As a matter of fact, none of the monitored alignments could be interpreted exactly as a normal or a parallel settlement trough. A shape function based on the complementary error function was thus proposed to fit the observed settlement data in the plane considering both the distances x and y respectively parallel and normal to the excavation^11^.

**Figure 22:**
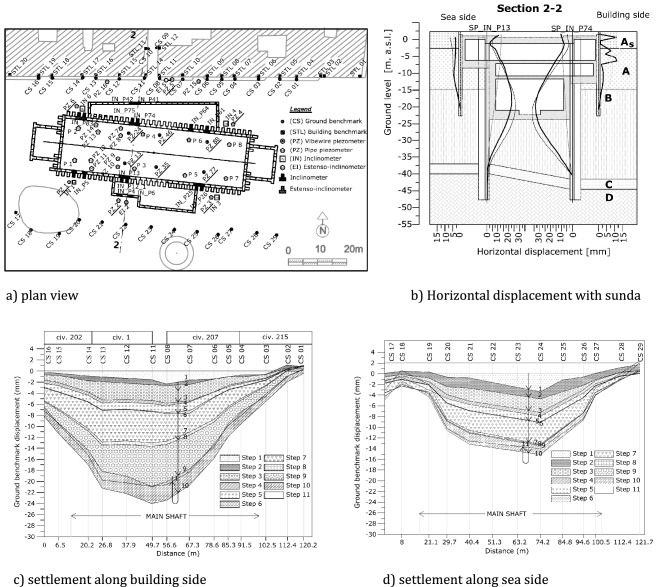
Plan view of the San Pasquale station site with measured longitudinal settlement profiles.

A plan view of the site is shown in Fig. 22a; the dimensions L = 85.5 m and B = 24.1 m of the excavation shaft are reported, and benchmarks installed for topographic survey are represented. The settlement profiles measured along the two alignments (i.e.: sea side and buildings side) are simply recalled in Fig. 22c,d. More details may be found in the paper by Russo et al.^11^. Along the sea side, the horizontal displacement profile of the diaphragm walls, reported in Fig. 22b, “predicts” a spandrel shape for the transversal profile which in turn should be compared with the prediction (Table 6) derived from the unpropped case. The values of $A$ and ∆ obtained by fitting the *quasi* longitudinal settlement profiles with the function $g\left(y\right)$ of Eq. (11) span respectively in the range 2 ÷ 6 m and in the range 22 ÷ 25 m. Along the building side, the horizontal displacement profile (Fig. 22b) “predicts” a concave shape for the transversal profile which should be compared to the prediction (Table 6) derived from the propped case. In such a case the fitting on the corresponding *quasi* longitudinal settlement profile indicates that $A$ ranges between 2 and 6 m whilst $\Delta$ between − 43 m and − 59 m. These values are simply plotted in the two Figs. 18 and 19 showing a good agreement with the FEM computed results and thus confirming the validity of the analytical relationship summarized in Table 6. It is here recalled for the convenience of the reader that for the *San Pasquale* station ${H}_{e}/L=0.3$ and $\lambda$ = 3.5.

## Conclusions

After a careful review of the existing literature on semi-empirical indications for separate 2D transversal and longitudinal surface settlement pattern around a deep excavation a new two variable function to describe the overall 3D settlement profile behind sides of a rectangular deep excavation has been built and discussed. That is a combination of two separate functions f(y) and g(x). The new two variables function depend on four independent shape parameters and one scale parameter which is the maximum settlement. As widely reported in the scientific literature while the maximum settlement depends on soil mechanical parameters the shape of the settlement trough is mainly dependent on geometry and constraints.

The quantitative relationships among the shape parameters and the main geometrical features of the excavation pit or the type of the supporting structural system (i.e., Propped or Unpropped) have been explored using settlement computed by solving several 3D FEM models inspired by typical and repetitive underground station boxes of the transportation network recently developed in the city of Napoli and embedded in pyroclastic sandy layers overlying the bedrock of the yellow tuff formation without groundwater table. The fitting toolbox by *Matlab* was used to carry out the least square regressions with the proposed new two variable function applied to the subsidence trough produced by Plaxis 3D and adequately sampled via a *Python* script.

The 3D surface fitted successfully on all the data sets from the cases computed via the FEM code showing high values of the R^2^ coefficient and a random distribution of the residuals. This success clearly indicates the potentiality of the function to be the main element on which to build a semiempirical method. In this paper several preliminary but sound indications on how to fix the shape parameters of the 3D function have been obtained and proposed, verifying, modifying, and integrating previous suggestions by Ou and Hsieh^25^ and Roboski and Finno^8^ separately provided for the 2D transversal and longitudinal settlement profiles. With regard to the transversal function f(y) it has been shown that:

i. the shape of the horizontal displacement profile of the diaphragm wall summarized by the ratio ${A}_{s}/{A}_{c}$ is a good predictor of the expected shape of the settlement profile at the ground surface, i.e. for ${A}_{s}/{A}_{c}>1.6$ the settlement profile will have a concave shape while for ${A}_{s}/{A}_{c}<1.6$ the settlement profile will have a spandrel shape;
ii. all the shape parameters of the transversal settlement profile, i.e. the slope ${f{^{\prime}}}_{0}$, the value of the function ${f}_{1}$ at the transition zone between PIZ and SIZ and the location of the maximum settlement ${y}_{m}$ at least for the concave shapes (nearly corresponding to the top propped cases) maybe defined relying upon the suggestions by Ou and Hsieh^25^ which compared to the FEM computations revealed either exact or at least conservative; the same does not hold true for the spandrel shapes (roughly corresponding to the top unpropped cases).
  Regarding the two shape parameters $A$ and $\Delta$ defining the longitudinal settlement function g(x) new sound indications have been summarized in analytical relationships involving the geometrical features H_e_/H_g_; H_e_/L; L/B of the excavation shaft. Previous indications by Roboski and Finno^8^ on the parameter A have been integrated and modified recognizing that:
iii. the value of A for all the analysed propped cases fall in the narrow range [0.1–0] while for all the analysed unpropped cases the range is [− 0.1 to 0]; for such a reason the parameter A can be set to 0 as first approximation when using the suggested empirical approach, while more detailed suggestions are available in Table 6;
iv. the value of $\Delta$ strongly depends on the geometrical features of the excavation and on the constraints (i.e. propped or unpropped cases) with preliminary indications on how to fix it summarized in Table 6.

The above findings obtained via a careful scrutiny of the FEM computed results have been applied on three case studies of excavations in sand where the settlements at the ground surface are only influenced by the retaining walls deformations, resulting in a general rather satisfactory and encouraging agreement. It is worthy to remark that case studies of excavations in sand with good quality monitoring data are by far rarer in the scientific literature and as a matter of fact most of the empirical methods recalled at the beginning of the paper are often oriented towards excavations in clay. This paper has also contributed to show that this distinction is not that important at least in the cases where the excavation in sand does not involve seepage inside the excavation shaft.

## Data Availability

The datasets generated during and/or analysed during the current study are available from the corresponding author on reasonable request.
